# Temporal changes in regulatory T cell subsets defined by the transcription factor Helios in stroke and their potential role in stroke-associated infection: a prospective case–control study

**DOI:** 10.1186/s12974-023-02957-w

**Published:** 2023-11-23

**Authors:** Maria Lukasik, Magdalena Telec, Radoslaw Kazmierski, Izabela Wojtasz, Natalia Andrzejewska-Gorczyńska, Wojciech Kociemba, Grzegorz Dworacki, Wojciech P. Kozubski, Magdalena Frydrychowicz

**Affiliations:** 1 Department of Neurology, Poznan University of Medical Sciences, Poznan, Poland; 2 Department of Neurology, Collegium Medicum, University of Zielona Gora, Zielona Gora, Poland; 3 Medicover, Poznan, Poland; 4 Department of Radiology, HCP Medical Center, Poznan, Poland; 5 Department of Immunology, Poznan University of Medical Sciences, Poznan, Poland

**Keywords:** Regulatory T cells, Transcription factor Helios, Stroke, Stroke-associated infection, Stroke immunology

## Abstract

**Background:**

Regulatory T cells (Tregs) are involved in the systemic immune response after ischemic stroke. However, their role remains unclear, and the effect appears to be both neuroprotective and detrimental. Treg suppressor function may result in immunodepression and promote stroke-associated infection (SAI). Thus we assume that the bidirectional effects of Tregs may be in part attributed to the intracellular transcription factor Helios. Tregs with Helios expression (H+ Tregs) constitute 70–90% of all Treg cells and more frequently than Helios-negative Tregs (H− Tregs) express molecules recognized as markers of Tregs with suppressor abilities.

**Methods and results:**

We prospectively assessed the circulating Treg population with flow cytometry in 52 subjects on days 1, 3, 10 and 90 after ischemic stroke and we compared the results with those obtained in concurrent age-, sex- and vascular risk factor-matched controls. At all studied time points the percentage of H+ Tregs decreased in stroke subjects—D1: 69.1% *p* < 0.0001; D3: 62.5% (49.6–76.6), *p* < 0.0001; D10: 60.9% (56.5–72.9), *p* < 0.0001; D90: 79.2% (50.2–91.7), *p* = 0.014 vs. controls: 92.7% (81.9–97.0) and the percentage of H− Tregs increased accordingly. In patients with SAI the percentage of pro-suppressor H+ Tregs on post-stroke day 3 was higher than in those without infection (*p* = 0.03). After adjustment for confounders, the percentage of H+ Tregs on day 3 independently correlated with SAI [OR 1.29; CI 95%: 1.08–1.27); *p* = 0.02]. Although the percentage of H+ Tregs on day 3 correlated positively with NIHSS score on day 90 (rS = 0.62; *p* < 0.01) and the infarct volume at day 90 (rS = 0.58; *p* < 0.05), in regression analysis it was not an independent risk factor.

**Conclusions:**

On the first day after stroke the proportion of H+ vs. H− Tregs changes in favor of pro-inflammatory H− Tregs, and this shift continues toward normalization when assessed on day 90. A higher percentage of pro-suppressive H+ Tregs on day 3 independently correlates with SAI and is associated positively with NIHSS score, but it does not independently affect the outcome and stroke area in the convalescent phase of stroke.

**Supplementary Information:**

The online version contains supplementary material available at 10.1186/s12974-023-02957-w.

## Introduction

T regulatory cells (Tregs) are involved in the systemic immune response after ischemic stroke. However, their role remains unclear and the effect appears to be both neuroprotective and detrimental. In some experimental studies it was found that Tregs reduce the area of damage [1–3], in others they had no effect [4, 5], while in some studies an unfavorable effect increasing the stroke area was confirmed [5, 6]. So far the exact site of action and the timing of activation have not been determined. The beneficial effect of Tregs is associated with their anti-inflammatory function and increased production of IL-10 [8, 9], TGFβ and IL-33 [3, 10], limiting the activation of T effector cells, and decreased production of pro-inflammatory IFN-γ [2, 9] and TNF-α [1, 3, 9]. Tregs diminish activation of microglia and macrophages [1, 3] and via IL-10 stimulate neurogenesis by the impact on neural stem cells that would migrate to the site of the injury and differentiate there into mature nerve cells, replacing the damaged ones [4]. However, the suppressor function of Tregs is a double-edged sword. Tregs have been proved to have long-term effects on stroke-induced immunodepression (SIID) by promoting IL-10 secretion. This unfavorable action exerts stroke-associated infection (SAI) and is responsible for significantly worse outcomes. Moreover, Tregs interact with platelets and endothelium and thus promote thromboinflammation and impede perfusion within the microcirculation as well as accelerating the death of neurons [11]. However, the inconsistent results of clinical studies on the role of Tregs in stroke encourage further research on their dual pro-inflammatory and suppressor nature.

Tregs are characterized by the markers CD4, CD25, and CD127 and the transcription factor FoxP3. The bidirectional function of Tregs might be in part assigned to the intracellular transcription factor Helios of the Ikaros family, discovered in the late 1990s. In physiological conditions Helios+ cells constitute 70–90% of the total Treg cell population and Helios+ (H+) and Helios− (H−) Tregs differ by about 1000 genes [12]. Helios seems to be important for the maintenance of Treg stability. Based on evidence in mice, H− Tregs downregulate Foxp3 and secondary to diminished activation of the STAT5 pathway upregulate the inflammatory cytokines IFN-γ, IL-2, and IL-17. In humans, studies of H+ vs. H− Tregs have yielded conflicting results, and until now it remains unclear to what extent Helios is involved in human Treg stability and function as well as what factors maintain Helios expression in Tregs [13, 14]. On the other hand, H+ Tregs more frequently than H− express CD103, Nrp-1, OX40, TNFRII and CD69—markers of activated Tregs with suppressor abilities. Nevertheless, it has been generally assumed that H+ Tregs are a pro-suppressor type and H− Tregs are a pro-inflammatory type [15].

Most research on the nature of Helios is experimental—in cell lines or animal models. There are only single clinical reports, and Helios expression in Tregs has not been studied in stroke patients. It seems that it may be related to the Janus face of Tregs during acute brain ischemia. The worse clinical outcome after stroke is in part a result of both autoimmunity and stroke-induced immunodepression. These two pathological processes may be the result of Treg function impairment, with the transcription factor Helios as the quarterback. Further elucidation of the factors that control Helios expression would help identify future therapeutic targets to manipulate Treg stability.

Taking into account the above considerations, we prospectively analyzed the population of circulating Tregs for Helios factor expression in acute, subacute and convalescent phases of stroke with special attention to patients developing SAI. We assumed that the Helios factor might play a role in the pro-suppressor phenotype of Tregs and in post-stroke immunodepression.

## Methods

### Study population

A total of 161 consecutive patients who presented with suspected stroke in the Stroke Unit at the Department of Neurology and Cerebrovascular Disorders of Poznan University of Medical Sciences, Poznan, Poland, between November 2018 and May 2019 were prospectively screened for inclusion in the study. Inclusion criteria were as follows: ischemic stroke, age over 40 and symptoms onset no more than 24 h prior to clinical evaluation and blood sampling. Following the consideration of the inclusion criteria 70 patients were prequalified for the study. The exclusion criteria were as follows: intracerebral hemorrhage, malignancies, autoimmune diseases, past/acute coronary syndrome and/or peripheral artery disease within 12 months preceding entry into the study, hematological disorders, liver and renal failure, alcohol and/or drug abuse and a history of infection and/or the use of antibiotics and/or immunosuppressants and/or steroids within the preceding 3 months. Subjects with transient ischemic attack (TIA) initially included in the study were excluded if the symptoms/signs resolved within 24 h and no lesions in magnetic resonance imaging (MRI) scanning with diffusion-weighted imaging (DWI) were visualized. Finally, 52 patients diagnosed with ischemic stroke, confirmed based on radiological evidence in cranial computed tomography (CT) scans and/or brain MRI at admission, were included in the study. The prequalified patients were not included because of intracranial hemorrhage (*N* = 9), TIA (*N* = 8) and active neoplasm (*N* = 1) (Additional file 1).

Clinical assessment was performed in the acute phase of stroke, on days 1 (D1), and 3 (D3), in the subacute phase on day 10 (D10) and 90 ± 3 days (D90) after stroke (convalescent phase). It included physical and neurological examination and the subjects were assessed on the National Institutes of Health Stroke Scale (NIHSS) and modified Rankin Scale (mRS). The etiology of the stroke was classified according to the TOAST classification [16] and location of the brain ischemia was established according to the Oxfordshire Community Stroke Project (OCSP) Classification (17). The standard diagnostic measures included blood pressure and height and weight assessments to calculate BMI. The clinical evaluation was supplemented by laboratory investigations including blood count with automatic smear test, coagulation, biochemical tests including C-reactive protein (CRP), urine tests, and electrocardiography (ECG), chest radiograph, color Doppler duplex ultrasonography, transcranial Doppler ultrasonography (TCD) and transthoracic ± transoesophageal echocardiography. The MRI scans were performed twice: within 24 h from the symptom onset and 90 ± 3 days after stroke. All stroke subjects were assessed for SAI on day 10 after stroke. SAI was defined as: (1) body temperature > 37.8 °C in a patient with symptoms suggestive of infection and/or (2) white blood cell (WBC) count > 11,000/mL or < 4000/mL and/or (3) inflammatory lesions in the chest X-ray and/or (4) blood or urine culture positive for pathogen and/or (5) antibiotic or chemotherapeutic therapy, all within 7 days from the onset of stroke symptoms.

The group of disease controls (DC) consisted of 34 age-, sex- and vascular disease risk factor-matched subjects who had never experienced any clinical symptoms of acute cerebral, coronary, or peripheral ischemia, but who were burdened with at least two vascular disease risk factors. Disease controls underwent the same laboratory tests and procedures as stroke patients except chest radiograph, neuroimaging, TCD and echocardiography. In this group blood sampling was performed once. Subjects with symptoms, signs and abnormalities in laboratory tests suggesting acute/chronic inflammation were not included in the study.

The study protocol was approved by the bioethics committee of the Poznan University of Medical Sciences (no. 139/2018). Informed consent was obtained from all study subjects.

### Flow cytometry

To detect the absolute number and percentage of circulating Tregs and H+ Tregs or H− Tregs, flow cytometry combined with counting was used. Blood samples in the stroke group were taken within 24 h of symptoms onset (D1), as well as 3 (D3), 10 (D10) and 90 ± 3 (D90) days after stroke and once in the DC group. Fasting venous blood was collected from the antecubital vein, directly into a BD Vacutainer tube (Becton Dickinson, USA) containing the anticoagulant K2EDTA (ethylenediaminetetraacetic acid dipotassium salt) at a concentration of 1.8 mg/mL of whole blood. The labeling procedures were performed within 4 h of blood collection. Cells were stained with combinations of the following antibodies (clone): anti-CD3/APC-Cy7 (UCHT1), anti-CD4/PE-Cy7 (SK3), anti-FOXP3/PE (150D), anti-Helios/APC (22F6), all from BioLegend, USA, anti-CD25/PerCP(MEM-181), Thermo Fisher Scientific, USA, and corresponding mouse IgG1, isotype controls. Test tubes with 20 µl of anti-CD3/CD4/CD25 antibodies mixed with 100 µl of peripheral blood were incubated at room temperature in the dark for 20 min. After this time, 500 µl of lysis buffer (FASC lysing solution, Becton Dickinson, USA) was added to each test tube and then incubated for 10 min. Lysis was stopped with phosphate-buffered saline solution (PBS, Roche Diagnostic, Germany). Samples were washed by spinning at 250 × g for 4 min. The supernatant was discarded. Samples were fixed and permeabilized. Saponin solution (BD Biosciences, USA) was added to each tube, mixed, and incubated at 4 °C in the dark for 60 min. Then, the cells were centrifuged and supernatant was removed. For intracellular staining of the transcription factor FOXP3 and Helios, 5 µl of anti-FOXP3/PE and 5 µl of anti-Helios/APC antibodies or isotype controls were added to appropriate tubes and incubated at room temperature in the dark for 60 min. Then, samples were washed and resuspended in 0.5 mL of saponin buffer for analysis with a flow cytometer. Data acquisition and analysis were performed using the FACSCanto II flow cytometry system and FACSDiva software (BD Biosciences) with a standard 6-color filter configuration. Lymphocytes were identified based on cell characteristic properties in the forward (FSC) and side (SSC) scatter. For additional analyses, gates were restricted to CD3^+^, CD3^+^ CD4^+^, CD3^+^ CD4^+^ CD25^+^ and CD3^+^ CD4^+^ CD25^high^ FOXP3^+^, Helios^+/−^ (18) (Additional file 2).

#### Infarct volume measurement

MRI sequences were performed on a Siemens Avanto 1.5-T clinical scanner. Images were blinded to patient identifiers. The standard imaging protocol covers sequences in FLAIR and T2-weighted images. Then diffusion-weighted image (DWI) sequencing with increasing b-factor and ADC maps was performed. The study ended with T1-weighted images in the axial plane. Hyperintense recent ischemic lesions were traced according to the segmentation method from DWI with b = 1000 mm/s^2^ by an expert reader (W.K.). The obtained surface area was multiplied by the thickness of the layers with the visualized ischemic region. Follow-up MR examination was performed after 90 ± 3 days and the volume of the stroke lesion was determined in FLAIR sequences in the same way. The lesion volume was expressed in mL. Typical imaging parameters were as follows: DWI: b = 0–500–1000–2000s/mm^2^, TR/TE ≈ 4700/113 ms; FLAIR: TR/TE ≈ 9000/86 ms; TI ≈ 2200 ms; FOV = 24 cm; matrix = 256 × 256; NEX = 1; and base resolution = 256, where TR indicates repetition time; TE, echo time; TI, inversion time; FOV, field of view; NEX, number of acquisitions. The concordance of the intrarater readings was 89.1% [κ = 0.76; 95% confidence interval (CI) (0.64–0.83) for DWI and FLAIR sequences.

### Statistical analyses

All data were analyzed using the data analysis software system Statistica, version 13 (TIBCO Software Inc. 2017) and GraphPad Prism, version 8.0.1 (GraphPad Software, San Diego, California). The sample size was evaluated a priori using standard statistical criteria for the estimation of sample size and statistical power. In order to reveal at least 30% difference between studied groups, with statistical power of at least 90%, and a significance of at least 1% taking into account that the ratio of case to control sample size equals 1, the estimated minimum sample size is 34 stroke subjects and 34 matched controls. The normal data distribution was tested with Shapiro–Wilk and Kolmogorov–Smirnov tests. Data showing a non-normal distribution are presented as median value and interquartile range values and were analyzed with non-parametric methods. Data with normal distribution were presented as means ± SD. The parametric Student’s t-test, ANOVA and post hoc Tukey test were used for comparisons of normally distributed data. Multiple comparisons within the stroke group were performed using the Wilcoxon signed-ranks test with Bonferroni correction; thus differences were assumed to be significant at *p* < 0.01. The Mann–Whitney U test was used to compare data between patients and the control group. Categorical data were compared by the Chi-squared test or Fisher’s exact test where appropriate. The Spearman rank correlation test was used to test for possible relationships between the studied parameters. Multivariate logistic regression analyses were used to identify independent risk factors for SAI, and poor outcome while multiple linear regression analysis was implemented to assess an independent association between Helios expression on Tregs and infarct volume. Where not indicated otherwise, a *p*-value < 0.05 was assumed to be significant.

## Results

The study population comprised 52 stroke subjects, 48% were female, and the mean age was 69 ± 12 years. The demographic and clinical characteristics are shown in Table 1. The patients were assessed on days 1, 3, 10 and 90. One patient died on day 7. On day 90, thirty-three subject were assessed—eight patients refused to participate in the last follow-up visit, 4 were unable to attend the department due to disability and dependency, 3 subjects were undergoing inpatient rehabilitation at that time and 3 patients had moved to another province. The controls were matched with the stroke patient according to the vascular disease risk factors, but atrial fibrillation was more prevalent in the stroke group (39% vs. 9%, *p* < 0.01) (Table 1).

**Table 1:** Baseline characteristics of studied groups

	Stroke patients D1 *N* = 52	Disease controls *N* = 34	*p* value
Age, years	69 (± 12)	68 (± 13)	0.66
BMI (kg/m^2^)	25.4 (23.6–28.4)	27.0 (25.1–31.1)	0.37
Females, *n* (%)	25 (48)	13 (38)	0.39
Hypertension, *n* (%)	42 (81)	30 (88)	0.56
Diabetes, *n* (%)	10 (19)	6 (18)	> 0.99
Ischemic heart disease, *n* (%)	19 (37)	14 (41)	0.82
Atrial fibrillation, *n* (%)	20 (39)	3 (9)	< 0.01
Hyperlipidemia (%)	16 (31)	12 (35)	0.81
Smoking, *n* (%)	18 (35)	7 (21)	0.23
Treatment, *n* (%)			
Thrombolysis	16 (31)	–	
Antiplatelet drugs	45 (87)	34 (100)	0.72
Anticoagulant	7 (14)	0	0.04
ACEI	20 (39)	17 (50)	0.37
Diuretics	23 (44)	12 (35)	0.51
CCB	22 (42)	17 (50)	0.51
β-blockers	9 (17)	8 (24)	0.58
ARB	7 (14)	1 (3)	0.14
Statins	14 (27)	15 (44)	0.11
Hypoglycemics	8 (15)	4 (13)	0.78
Insulin	3 (6)	3 (9)	0.68
Stroke etiology (TOAST classification), *n* (%)			
LVD	13 (25)	–	–
SVD	12 (23)	–	–
CE	22 (42)	–	–
OE	5 (10)	–	–
UE	0	–	–
Stroke location, *n* (%)			
TACI	6 (12)	–	–
PAC	22 (42)	–	–
POCI	13 (27)	–	–
LACI	10 (8)	–	–
Stroke lesion volume, mL			
Day 1	2.1 (0.3–11.2)	–	–
Day 90	1.0 (0.4–4.7)	–	–

*ACEI* angiotensin convertase enzyme inhibitors, *CCB* calcium channel blockers, *ARB* angiotensin receptor blockers, *LVD* large vessel disease, *SVD* small vessel disease, *CE* cardioembolic stroke, *OE* stroke of other etiology, *UE* stroke of unknown etiology, *TACI* total anterior circulation infarcts, *PACI* partial anterior circulation infarcts, *POCI* posterior circulation infarcts, *LACI* lacunar infarcts

**Fig. 1:**
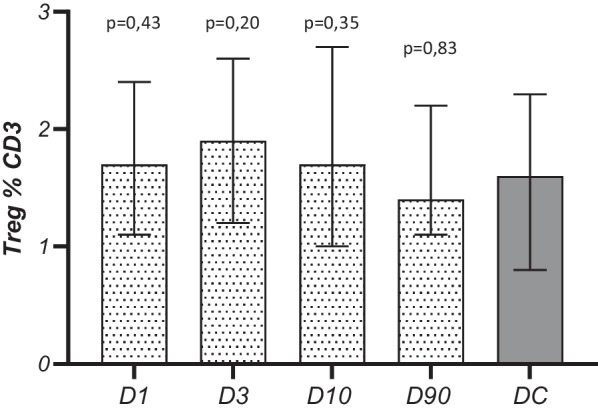
Temporal pattern of Tregs (%) in subjects after stroke and disease controls

The median time from stroke onset to blood sampling on D1 was 17 h (12–22). The peripheral blood leukocyte count (WBC), the absolute number and the percentage of Tregs in the population of lymphocytes, defined as CD3+ cells, did not change significantly in the time after the stroke, although they were the highest (non-significant) on post-stroke day 3. The values did not differ significantly from those observed in the control group either (Table 2, Fig. 1). The absolute number and the percentage of lymphocytes on post-stroke days 1, 3, and 10 were lower than those observed in controls, and on day 1 the percentage of lymphocytes was significantly lower than that measured on day 90 (Table 2).

**Table 2:** Prospective quantitative analysis of white blood cell subpopulations and C-reactive protein in stroke patients and controls

	Stroke D1 *N* = 52	Stroke D3 *N* = 52	Stroke D10 *N* = 51	Stroke D90 *N* = 33	Disease control group *N* = 34	p D1 vs. DC	p D3 vs. DC	p D10 vs. DC	p D90 vs. DC
WBC × 10^3^/µl	7.4 (5.8–10.6)	7.6 (5.7–10.2)	7.2 (5.9–8.1)	6.8 (5.6–7.7)	6.8 (5.7–8.4)	0.12	0.28	0.82	0.45
Lymphocytes × 10^3^/µl	1.7 ± 0.9	2.0 ± 0.9	1.8 ± 0.7	1.9 ± 0.7	2.3 ± 1.2	**0.008**	**0.01**	**0.02**	0.07
Lymphocytes %	21.5 ± 11.6*	25.3 ± 10.9	25.8 ± 10.2	28 ± 7.5	31.3 ± 10	**< 0.0001**	**0.02**	**0.03**	0.16
Treg (cells/μl)	13.2 (8.6–19.8)	15.4 (9.2–27.4)	12.9 (8.7–18.2)	14.5 (10.2–20.7)	16.4 (9.07–21.1)	0.61	0.62	0.42	0.91
Treg H+ (cells/μl)	8.0 (4.9–15.8)	9.1 (4.0–21.2)	6.1 (4.6–12.9)	9.4 (7.5–15.9)	13.8 (7.1–18.6)	**0.03**	**0.04**	**0.03**	0.27
Treg H− (cells/μl)	4.6 (2.6–6.81)	4.7 (3.5–9.8)	5.3 (3.9–7.3)	4.3 (1.0–8.5)	1.0 (0.5–3.1)	**< 0.0001**	**< 0.0001**	**< 0.0001**	**0.006**
H−/H+ Treg ratio	0.5 (0.3–0.9)	0.6 (0.4–1.0)	0.6 (0.5–0.8)	0.2 (0.1–1.0)	0.05 (0.02–0.15)	**< 0.0001**	**< 0.0001**	**< 0.0001**	**0.001**
CRP, mg/L	4.2 (1.8–9.9)*	6.1 (2.1–22.1)*	3.8 (1.2–19.2)*	1.9 (0.4–5)	1.2 (0.6–2.2)	**0.001**	**< 0.0001**	**0.03**	0.23

*p*-values in bold are statistically significant

*Difference significant in relation to the value observed on D90 (*p* < 0.001). No other significant differences between D1-D3-D10-D90 were observed

### Kinetics of circulating Tregs

**Fig. 2:**
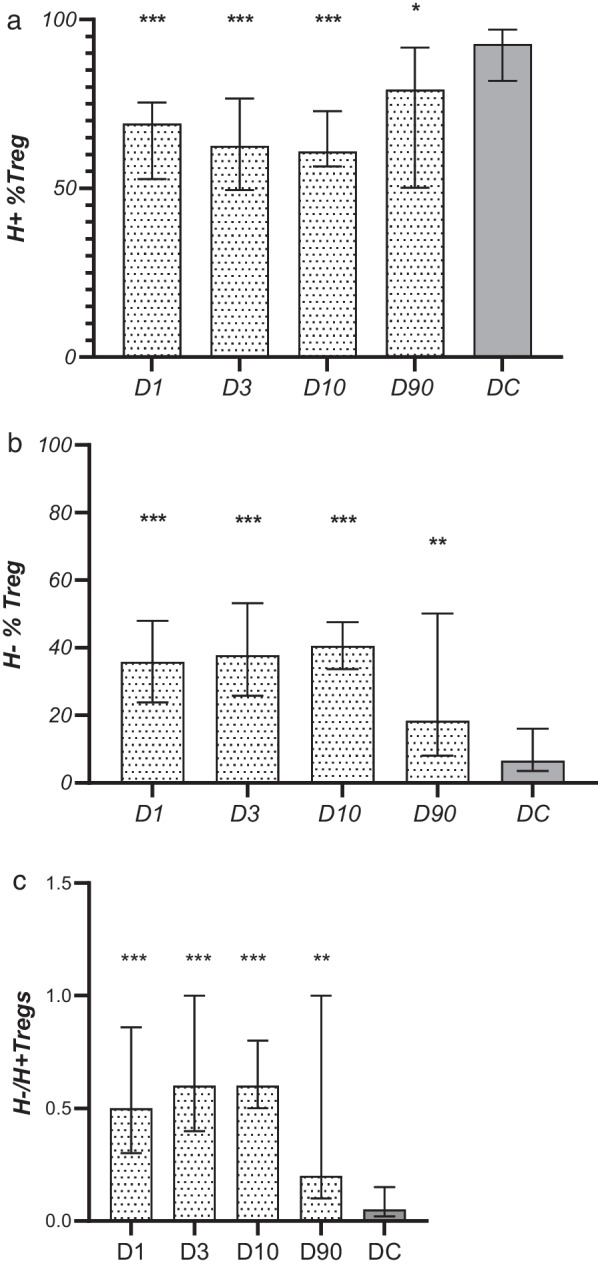
Temporal pattern of **a** Helios+ (H+) Tregs, **b** Helios− (H−) Tregs subpopulation **c** H−/H+ Treg ratio in patients and disease controls (DC). The significance of differences between stroke subjects and DC is indicated as follows: *p < 0.05; **p < 0.01; ***p < 0.001. No statistically significant differences in the stroke group between D1-D3-D10-D90 were observed

The percentages of H+ Tregs on post-stroke days 1, 3, 10, and 90 were significantly lower than those observed in the control group [D1: 69.1% (52.7–75.4), *p* < 0.0001; D3: 62.5% (49.6–76.6), *p* < 0.0001; D10: 60.9% (56.5–72.9), *p* < 0.0001; D90: 79.2% (50.2–91.7), *p* = 0.014 vs. controls: 92.7% (81.9–97.0); Fig. 2a] and the absolute number of circulating H+ Tregs on days 1, 3 and 10 was lower as well (Table 2). As expected, both the absolute number and the percentage of H− Tregs on post-stroke days 1, 3, 10, and 90 were significantly higher than the values observed in controls [D1: 35.8% (23.9–48.0), *p* < 0.0001; D3: 37.8% (25.8–53.2), *p* < 0.0001; D10: 40.5% (33.7–47.6), *p* < 0.0001; D90: 18.4% (8.0–50.2), *p* = 0.003 vs. controls: 6.5% (3.5–16.0); Fig. 2b, Table 2]. In stroke patients the percentages of H+ and H− Tregs did not differ between studied time points, although a non-significant gradual decrease of the H+ Treg population up to day 10 and a non-significant gradual increase of the H− Treg population up to day 10 were observed. Despite the tendency for the H−/H+ Treg proportions to normalize on day 90, these values did not significantly differ from those observed in acute and subacute phases of stroke.

On D1 the percentage of H+ Tregs was significantly higher in male than in female stroke patients [respectively: 74.7% (61.5–86.8) vs. 61.5% (46.7–71.5); *p* = 0.004], while in females on D1 the percentage of H− Tregs was significantly higher than in males [respectively: 37.5% (30.4–52.0) vs. 27.4% (17.3–45.9); *p* = 0.037]. Such a difference was not found in the following days of observation or in the disease control group.

### Prospective clinical evaluation with regard to circulating Tregs and subsets

#### Stroke-associated infection

The criteria of SAI were met by 25 patients (48%). Twenty patients were SAI+ as early as day 3. Between days 3 and 7 after stroke, another 5 patients developed SAI (6 patients developed SAI on day 2, 14 on day 3, 2 on day 4, 2 on day 5, and one patient on day 6). The average time from stroke onset to the SAI symptoms was 3 ± 1 days. The most common infection was pneumonia (14 patients; 56%), followed by urinary tract infection (9 patients; 36%) and other infections (2 patients; 8%). The number of subjects treated with antibiotics was as follows: on D2, 4 patients; on D3, 20 patients; and on D10, 25 patients. The baseline characteristics of SAI+ and SAI− patients are shown in Table 3.

**Table 3:** Comparative characteristics of SAI+ and SAI− patients

	SAI+ *N* = 25	SAI− *N* = 27	*p* value
Age, years	73 ± 13	66 ± 12	0.24
Sex M/F, *n*	12/13	14/13	0.76/0.83
Time from stroke onset to blood sampling on D1, hours	18.5 (12–22)	17 (12–21)	0.57
CRP D1, mg/L	7.5 (6.1–27.5)	3.6 (1.3–6.4)	**0.004**
CRP D3	17.3 (7.9–69.5)	3.5 (1.2–6.5)	**< 0.001**
CRP D10	20.7 (2.8–45.1)	2.5 (1.3–5.1)	**0.002**
CRP D90	3.5 (1.4–5.3)	2.3 (1.1–3.8)	0.21
WBC D1, × 10^3^/µl	10.6 ± 3.6	7.6 ± 2.5	**< 0.0001**
WBC D3	10.7 ± 5.3	6.8 ± 1.6	**< 0.00001**
WBC D10	9.1 ± 3.9	6.7 ± 1.5	**0.002**
WBC D90	7.1 ± 2.0	7.0 ± 0.9	0.63
Lymphocytes D1, × 10^3^/µl	2.2 ± 1.1	1.8 ± 0.6	0.57
Lymphocytes D3	1.5 ± 0.8	2.4 ± 1.7	**0.01**
Lymphocytes D10	1.7 ± 0.9	1.9 ± 0.6	**0.04**
Lymphocytes D90	1.9 ± 0.8	1.9 ± 0.6	0.56
Lymphocytes D1, %	17.0 ± 10.2	25.4 ± 8.6	**< 0.001**
Lymphocytes D3	16.8 ± 6.9	27.7 ± 8.7	**< 0.0001**
Lymphocytes D10	19.1 ± 10.4	27.4 ± 8.7	**0.006**
Lymphocytes D90	27.1 ± 7.3	28.9 ± 8.4	0.91
H+ %Treg D1, %	73.7 (61.3–75.7)	69.4 (52.7–75.4)	0.76
H+ %Treg D3	76.6 (58.3–87.7)	58.8 (48.3–71.2)	**0.03**
H+ %Treg D10	59.6 (48.1–64.6)	65.1 (58.1–74.4)	0.11
H+ %Treg D90	88.7(64.1–99.8)	49.7 (43.0–89.4)	0.66
H+/H− D1	0.46 (0.3–1.3)	0.6 (0.37–0.9)	0.82
H+/H− D3	0.45 (0.15–0.85)	0.75 (0.4–1.3)	**0.04**
H+/H− D10	0.6 (0.5–0.8)	0.7 (0.5–1.1)	0.92
H+/H− D90	0.4 (0.11–1.2)	0.3 (0.1–1.0)	0.97
NIHSS D1, pts	9 (5–19)	3 (1–6)	**0.001**
NIHSS D3	13 (5–25)	2 (1–4)	**< 0.001**
NIHSS D10	13 (2–20)	1 (0–2)	**< 0.001**
NIHSS D90	4 (2–6)	1 (0–3)	0.15
mRS D3, pts	4 (1–5)	1 (0–3)	**< 0.001**
mRS D10	4 (1–5)	1 (0–2)	**< 0.001**
mRS D90	1 (0–3)	0 (0–1)	0.05
Stroke volume D1, mL	5.6 (0.2–25)	2.8 (0.5–9.5)	0.45
Stroke volume D90	1 (1–25)	1 (0.1–2.7)	0.76

*p*-values in bold are statistically significant

WBC—white blood cells; lymphocytes—percentage of lymphocytes in leukocyte population (automatic blood smear); Treg%CD3—percentage of Tregs in lymphocyte population (flow cytometry); H+ %Treg—percentage of Tregs with Helios expression in Treg population (flow cytometry); NIHSS—National Institutes of Health Scale Score; mRS—modified Rankin Scale score; D1, D3, D10, D90—days after stroke

**Fig. 3:**
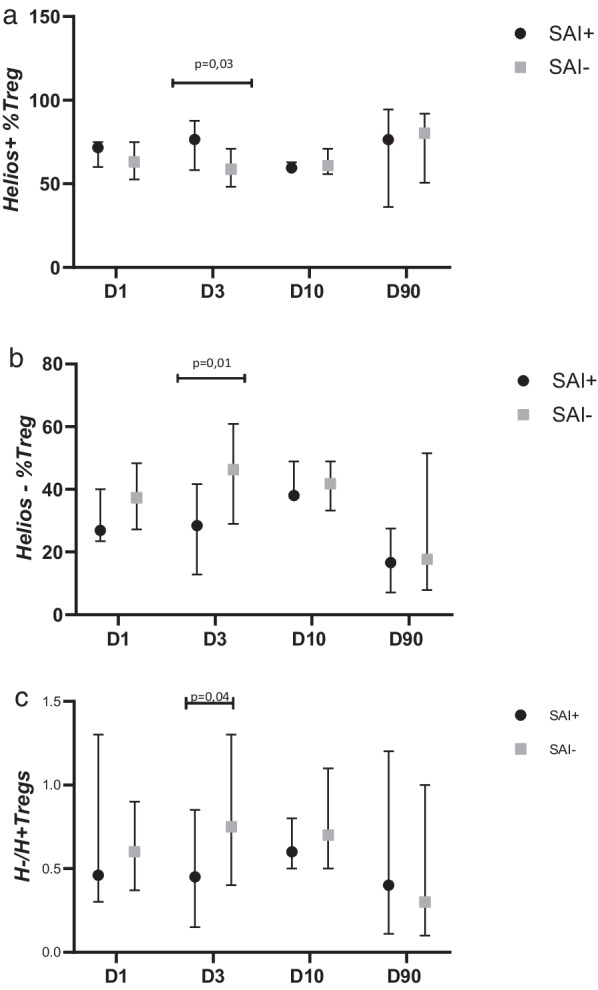
Percentage of **a** Helios+ (H+) Tregs, **b** Helios− (H−) Tregs in population of Tregs and **c** H−/H+ Treg ratio in patients with and without stroke-associated infection (SAI)

There were no differences between SAI+ and SAI− subjects in the percentage and the absolute number of circulating Tregs at any time point after stroke. However, on D3, patients with SAI as compared to those without SAI showed a significantly higher percentage of H+ Treg and a significantly lower percentage of H− Treg [H+ Treg: 76.6% (58.3–87.7) vs. 58.8% (48.2–71.2), *p* = 0.03 and H− Treg: 28.4% (12.8–41.2) vs. 46.3% (29.3–60.9), *p* = 0.01] (Fig. 3).

No significant differences between studied time points within the SAI+ or SAI− group were found. To demonstrate whether H+ %TregD3 is independently associated with SAI, we built a multiple regression model (Table 4). We entered in the model the independent variables that significantly differed between the SAI+ and SAI− groups and other established factors that significantly correlate with SAI, such as age, male sex, volume of the stroke lesion or clinical condition assessed on the NIHSS on the first day of stroke [19]. In multivariate logistic regression analysis models including as independent variables age, sex, neurological status on D1 (NIHSS score), infarct volume on D1, and selected leukocyte subpopulations, SAI was independently and positively associated with WBC and percentage of H+ Tregs, both on D3, but not with the Treg percentage. A negative association between SAI and smear lymphocyte percentage of borderline significance was observed (OR = 0.94, *p* = 0.05) (Table 4). SAI was not found to correlate with parameters assessed on D1 and D10: WBC, lymphocytes%, Treg% and H+/H− % Treg.

**Table 4:** The associations between SAI as the dependent variable and other risk factors for poor outcome

Model/*p*-value for model	Age	Male sex	NIHSS D1	Ischemic lesion volume D1	Added variable: leukocytes/leukocyte subset
	OR (95% CI); *p*-value				
Model 1 *p* < 0.001	0.99 (0.97–1.03); 0.99	1.12 (0.45–2.77) 0.79	**1.36** (1.28–1.46) ** < 0.001**	**1.21** (1.18–1.21) **0.03**	–
Model 2 *p* < 0.001	1.01 (0.96–1.04); 0.76	1.04 (0.39–2.77) 0.92	**1.32** (1.22–1.43) **0.008**	**1.21** (1.17–1.23) **0.04**	**WBC D3** **1.64** (1.34–2.01) **0.001**
Model 3 *p* < 0.001	0.99 (0.95–1.03); 0.74	1.16 (0.46–2.92) 0.75	**1.33** (1.24–1.43) **0.003**	**1.21** (1.17–1.24) **0.04**	Lymphocytes % D3 0.94 (0.89–1.00) 0.05
Model 4 *p* < 0.001	0.68 (0.1–4.48); 0.69	1.09 (0.58–2.05) 0.79	**1.37** (1.28–1.47) ** < 0.001**	**1.21** (1.18–1.24) **0.04**	Treg%CD3 D3 0.67 (0.32–1.39) 0.28
Model 5 *p* < 0.001	0.99 (0.96–1.03); 0.84	1.15 (0.46–2.87) 0.76	**1.34** (1.28–1.46) **0.001**	**1.21** (1.18–1.24) **0.04**	**H+ % Treg D3** **1.29** (1.08–1.27) **0.02**

Multivariate logistic regression models describe the associations between stroke-associated infection (SAI) as the dependent variable and other risk factors for poor outcome including immune response cells

Values in bold are statistically significant

WBC—white blood cells; lymphocytes—percentage of lymphocytes in leukocyte population (automatic blood smear); Treg%CD3—percentage of Tregs in lymphocyte population (flow cytometry); H+ %Treg—percentage of Tregs with Helios expression in Treg population (flow cytometry); NIHSS—National Institutes of Health Scale Score; D1, D3—days after stroke

#### Neurological deficit and infarct volume

The lowest median NIHSS score was observed on D1 and was 7 (4–11). The NIHSS score was significantly higher (worse clinical outcome) in SAI+ patients at post-stroke days 1, 3 and 10. On D90 the neurological status did not differ between SAI+ and SAI− groups.

The percentage of Tregs in the lymphocyte population at all studied time points positively correlated with NIHSS score (the more Tregs, the worse the neurological condition). On D3 H+ Tregs% correlates positively and H− Tregs% negatively with NIHSS score and mRS score on D90 (Table 5).

**Table 5:** Correlations between neurologic and functional status and Treg populations

	NIHSS D1		NIHSS D3		mRS D3		NIHSS D10		NIHSS D90		mRS D90	
	rS	*p*	rS	*p*	rS	*p*	rS	*p*	rS	*p*	rS	*p*
Treg%CD3 D1	**0.51**	< 0.001	**0.37**	< 0.01	**0.31**	< 0.05	**0.31**	< 0.05	**0.43**	< 0.05	**0.31**	< 0.05
H+ %Treg D3	0.06	ns	0.23	ns	0.23	ns	0.19	ns	**0.62**	< 0.01	**0.40**	< 0.05
H− %Treg D3	− 0.18	ns	− 0.22	ns	0.29	ns	− 0.23	ns	− **0.53**	< 0.05	− **0.34**	< 0.05

Values in bold are statistically significant

Treg%CD3—percentage of Tregs in lymphocyte population; H+ %Treg—percentage of Tregs with Helios expression in Treg population; NIHSS—National Institutes of Health Scale Score; mRS—modified Rankin Scale score; D1, D3, D10, D90—days after stroke

However, in multivariate logistic regression analysis H+ %Tregs on D3 was not an independent risk factor for a poor outcome on D90 defined as mRS > 2 or death, while this was the case with other, well-established risk factors of functional impairment including age and neurological status on day 1 of stroke (Table 6). There were no correlations between studied Treg subsets on D10 and D90 and NIHSS score on D1–D90.

**Table 6:** The associations between poor outcome on day 90 and other risk factors for functional impairment

Model/*p*-value for model	Age	NIHSS D1	Added variable:
	OR (95% CI); *p*-value		
Model 1 *p* < 0.001	**1.06** (1.01–1.12); **0.02**	**1.71** (1.36–2.15) **< 0.001**	**–**
			SAI
Model 2 *p* < 0.001	**1.06** (1.01–1.11); **0.03**	**1.69** (1.33–2.13) **< 0.001**	**3.39** (1.00–12.19) **0.04**
			WBC D3
Model 3 *p* < 0.001	**1.06** (1.01–1.11); **0.03**	**1.70** (1.35–2.15) **< 0.001**	1.03 (0.78–1.38) 0.79
			H+ %Treg D3
Model 4 *p* < 0.001	**1.06** (1.01–1.11); **0.03**	**1.71** (1.35–2.15) **< 0.001**	1.01 (0.95–1.05) 0.84

Multivariate logistic regression model describes the associations between poor outcome on day 90 as the dependent variable and other established/potential risk factors for functional impairment

Values in bold are statistically significant

NIHSS—National Institutes of Health Scale Score; SAI—stroke-associated infection, WBC—white blood cells, H+ %Treg—percentage of Tregs with Helios expression in Treg population; D1, D3—days after stroke

Median infarct volume on D1 was greater than that on D90 (*p* < 0.001, Table 1). There was no difference between infarct volume in SAI+ and SAI− on D1 or D90 (Table 3), or between infarct volume on D1 and D90 in SAI+ subjects (*p* = 0.22) while in SAI− patients the lesion volume was significantly smaller on D90 (*p* = 0.0001).

Moreover, H+ Tregs% on D3 and D10 correlated significantly with the final infarct volume assessed in MRI on D90 after stroke (Table 7).

**Table 7:** Correlations between infarct volume and percentage of Tregs with/without Helios factor expression

	H+ %Treg D1	H− %Treg D1	H+ %Treg D3	H− %Treg D3	H+ %Treg D10	H− %Treg D10	H+ %Treg D90	H− %Treg D90
Infarct volume D1	0.49 ns	− 0.44 ns	0.49 ns	− 0.47 ns	0.54 ns	− 0.47 ns	− 0.16 ns	− 0.02 ns
Infarct volume D90	0.35 ns	− 0.34 ns	**0.58** *p* < 0.05	− 0.53 ns	**0.59** *p* < 0.05	− 0.43 ns	− 0.01 ns	− 0.11 ns

Values in bold are statistically significant

H+ %Treg—percentage of Tregs with Helios expression in Treg population; H− %Treg—percentage of Tregs without Helios expression in Treg population, D1, D3, D10, D90—days after stroke

Nonetheless, in multiple linear regression analysis, in the model including independent variables: H+ Treg% on D3, age and NIHSS on D1, the infarct volume on D90 as a dependent variable was not associated with the percentage of H+ Tregs on D3 (*p*-value for model = 0.24; R^2^ = 0.14).

There were no correlations between the percentage of Tregs on D1-90 and infarct volume (D1 and D90).

## Discussion

The role of Treg lymphocytes in ischemic stroke is still unresolved, despite a growing number of studies. Are their presence and action beneficial to the course of a stroke, or are Tregs a double-edged sword? [11]. Nevertheless, the factors that determine which face of Tregs we observe in stroke need to be currently defined. Notably, most of the research is conducted on rodents. Unfortunately, the translation of these results into the human brain is limited, and none of the immunotherapies that have been effective in animals has had the expected effects in humans.

In the present study, no differences were found between the absolute number and percentage of Treg cells in the lymphocyte population (CD3+) in post-stroke patients compared to controls. We also did not observe any change in the studied parameters in time. However, an insignificant increase in the percentage of Tregs was observed on the third day, when the Treg value was highest. It was consistent with the results obtained in experimental studies [20, 21] and prospective clinical stroke studies conducted by Santamaría-Cadavid et al. and Jiang et al. [22, 23]. In addition, Hein et al. also confirmed in nonseptic shock—a different but sudden, life-threatening condition—the highest number of circulating Tregs on day 3 [24]. Not all clinical findings are unequivocal, and several studies have reported a reduction in Tregs in the CD4+ lymphocyte population in patients with acute ischemic stroke [25–27]. These discrepancies may be due to methodological differences (different definitions of Treg subpopulations), heterogeneous control groups (control groups consisting of healthy volunteers or patients with acute vascular diseases such as TIA and myocardial infarction), and biological diversity of Tregs.

The most important finding of our study, however, is that for the first time we described a significant, gradual (at least until day 10) decrease in the percentage of circulating Helios+ Tregs in the acute and subacute phases of stroke and a concomitant successive increase in both absolute values and the rate of Helios− Tregs on day 90 when these differences were less pronounced, although still significant. Due to the gap interval between examinations on day 10 and day 90, it is unclear at what time point these values began to normalize.

Such a marked shift in the H− and H+ Treg ratio is apparent as early as day 1 after the stroke. So far, such early quantitative changes in Tregs have not been observed. Thus, we suppose that changes in the ratio of H−/H+ Tregs in favor of H− precede changes in the number of Tregs. Simultaneously we did not observe significant differences in Treg kinetics and only observed a direction of changes consistent with those found in studies on larger populations. Then we can presume that such marked differences in the H−/H+ Treg ratio may be a sensitive marker of immune changes in acute stroke. Since the kinetics of H−/H+ Tregs in emergency conditions have not been studied, we cannot relate our results to other studies.

H+ and H− Tregs are currently under research, and their phenotypic and functional differences are not definitively known. Gene expression analysis showed that the two populations differ by approximately a thousand genes. H+ Tregs have more surface antigens characteristic for activated Treg lymphocytes with suppressor properties, while the H− Treg subpopulation secretes more IFN-γ. Then it is assumed that H+ Tregs play a primary suppressor function, while the H− Treg subpopulation is pro-inflammatory. Interestingly, H+ Tregs are produced from virgin cells, but under lymphopenic conditions, they may assume a phenotype with low Helios expression, and they may become less stable and lose FoxP3 [28]. Lymphopenia is a feature of the acute phase of stroke, so the shift between H+ and H− Treg subpopulations in this study confirms Treg lymphocytes’ behavior observed in other clinical situations.

It seems that the H−/H+ Treg ratio may be of clinical significance. We observed that on the third day after the stroke, patients who developed SAI had a significantly higher percentage of H+ Tregs and a considerably lower percentage of H− Tregs than SAI− subjects. This finding is consistent with the fact that the down-regulation of IFN-γ (preferentially secreted by H− Tregs) increases the prevalence of SAI [29]. Moreover, when adjusting for confounders that are significant for SAI (inter alia, severity of initial neurologic deficit, WBC on day 3, and infarct volume), the higher percentage of H+ Tregs on day 3 remains an independent risk factor of SAI. In longitudinal observation, we did not find any significant differences in the ratio of H+ and H− Tregs between studied time points within both SAI+ and SAI− groups. However, we noted on day 3 an insignificant increase of both: the H+ Tregs in SAI+ and H− Tregs in SAI− subjects. Most patients developed SAI as early as day 3, consistent with the previous finding that stroke-associated pneumonia (SAP) usually occurs on days 2–3 [19]. Since an increased percentage of H+ Tregs coincides with SAI, one cannot assign a predictive value. However, it is worth considering that an increased rate of circulating pro-suppressive H+ Tregs may be one of the etiological factors for SAI. Nevertheless, we found no differences between SAI+ and SAI− subjects in the percentage and the absolute number of circulating Tregs at any time point after stroke. According to our best knowledge, up to now the association between Treg cells and post-stroke immunodepression or infections has not been confirmed in humans although there are theoretical premises supporting this thesis [30, 31]. Since the correlation between H+ %Treg on D3 and SAI was confirmed in our study, we suppose that the transcription factor Helios with its suppressor function might be crucial and the changes in the number of Tregs might be less important than the qualitative shift (lost Helios expression) within a quantitatively stable population.

Although an association between a higher percentage of circulating H+ Tregs in the acute phase of stroke and a worse clinical condition in the convalescent stage as well as with final infarct volume were described, these correlations were not confirmed in multivariate logistic regression analysis. Differences in the immune regulatory response depending on the severity of the clinical symptoms of stroke have been noted before [32]; however, the results varied. Neither Urra et al. [25] nor Ruhnau et al. [26] found a correlation between the neurological deficit and the number of Tregs. In contrast, Santamaría-Cadavid et al. found that an increased level of circulating Tregs within 48 h after stroke was associated with better functional status [33]. Such a discrepancy may be due to different methodologies implemented by the Santamaría-Cadavid group, while in the study by Urra et al., patients with symptoms of infection were excluded, which made the results non-comparable. And finally, in the context of our results, the clinical outcome seems to be a weak correlation, if any.

On the first day after stroke (but on other days not), we observed a larger decrease in H+ Tregs in females than in males. Such a pro-inflammatory shift in females is of unknown clinical significance and might be random, especially since we observed this phenomenon only on the first day of stroke. On the other hand, it might exert a detrimental effect since it is well documented that women with stroke are at risk of worse clinical outcomes. Moreover, the limited suppressive function of Tregs and bias toward pro-inflammatory ones are observed in many autoimmune diseases characterized by female prevalence. This phenomenon in premenopausal women is related to estrogen E2. Its normal level suppresses the expression of FoxP3—the marker and major transcription factor of Tregs and increased secretion of anti-inflammatory IL-10 by Tregs was seen [34, 35]. It was associated with poorer recovery and post-stroke immunosuppression. However, the biology of Tregs in postmenopausal women seems to be more complex and remains unclear [36].

A disturbed H−/H+ ratio is not the only early marker of acute dysfunction detected within the first 24 h of symptoms onset, which raises the eternal “chicken or egg” causality dilemma. As a transcription factor, Helios probably reacts rapidly to environmental changes; however, the nature of Helios is not sufficiently well known to unequivocally state that its expression is the consequence of ischemia. It seems that very accurate Treg kinetics in the first minutes or hour in stroke (and even more so in TIA) might shed new light into processes preceding clinical symptoms of brain ischemia. Such clinical studies are strongly needed. Nevertheless, we realize that their logistics is troublesome.

Since stroke-induced immunodepression needs to be a better-understood phenomenon, even preliminary data are valuable. There is no information on changes in the expression of the Helios marker in Treg lymphocytes in the stroke literature. In this matter, the present study should be considered pioneering. It seems to be an important voice in the discussion about the Janus face of Treg lymphocytes in the acute phase of stroke, with arguments in favor of their damaging function and the role of Helios as a quarterback. This variable proportion between H− and H+ Tregs reflects the disturbed systemic balance between pro-suppressive and pro-inflammatory processes that “fight” after a stroke and might significantly hinder therapy. Nonetheless, several mechanisms of immunosuppressive and, to a lesser degree, the pro-inflammatory function of Tregs have been identified, and the Helios factor is only one of them. At this stage of the research, we can only assume a scenario in which lymphopenia is present from the first day of stroke (Table 2), and creates a microenvironment promoting destabilization of Tregs. From the first day of stroke the H− /H+ Treg shift is present as well, resulting in an increased percentage of pro-inflammatory H− Tregs. However, on the 2nd/3rd day, in some patients, the shift toward pro-inflammatory H− Tregs is not so prominent; pro-suppressor H+ Tregs predominate, and patients in this group develop SAI. Such an unfavorable shift was not confirmed on day 10 and beyond.

Further elucidation of the factors that control Helios expression would help identify potential therapeutic targets to manipulate Treg stability. For example, depletion of Helios expression in a selected patient population may help to avoid stroke-induced immunodepression, and in another one, enhancement of Helios expression may reduce post-stroke autoimmunity. Nevertheless, until now the experimental studies on animal immunotherapeutic models have failed [37].

There are many limitations of our research. Firstly, this is a single-center study with a limited sample size. Despite the initial group size estimation, a larger group would undoubtedly allow more relationships to be identified. Secondly, conducting observations in the subacute phase of stroke only up to day ten makes it impossible to clarify the time the H−/H+ Treg ratio begins to normalize and whether this is of prognostic significance. Thirdly, we assessed the expression of Helios in Tregs using cytometry as the only method, and our data are not supported indirectly with other strategies (the concentration of IFN-γ or IL-10 and TGF-β, which both promote the differentiation of Tregs [37]) confirming that higher expression of Helios translates into Treg suppressor function. And finally, there was some bias due to the limited number of patients on day 90. Most dropped-out subjects could not participate in the study due to disability; thus, we could not assess the patients with the most unfavorable outcomes. Nevertheless, we believe that the above observations are pioneering and wish to sow the seeds for further research into the involvement of immune cells in the pathophysiology and perhaps even the pathogenesis of stroke.

## Conclusions

Early after stroke, the proportion of H+ vs. H− Tregs changes in favor of the pro-inflammatory H− Tregs, and this shift continues toward normalization when assessed on day 90. The percentage of pro-suppressor H+ Tregs on day 3 independently correlates with SAI and is associated positively with NIHSS score, but it does not independently affect the outcome and stroke area in the convalescent phase of stroke.

### Supplementary Information

**Additional file 1: **Flowchart of participant recruitment.

**Additional file 2: **Flow cytometry gating strategy.

## Data Availability

All data used and analyzed for the current study are available from the corresponding author on reasonable request.

## Abbreviations

Tregs: Regulatory T cells
SAI: Stroke-associated infection
H+ Tregs: Regulatory T cells with Helios expression
H− Tregs: Regulatory T cells without Helios expression
NIHSS: National Institutes of Health Stroke Scale
SIID: Stroke-induced immunodepression
IL: Interleukin
TGFβ: Tumor growth factor beta
IFN-γ: Interferon gamma
TNF-α: Tumor necrosis factor alpha
CD: Cluster of differentiation
TIA: Transient ischemic attack
MRI: Magnetic resonance imaging
CT: Computed tomography
OCSP: Oxfordshire Community Stroke Project
CRP: C-reactive protein
BMI: Body mass index
ECG: Electrocardiography
TCD: Transcranial Doppler ultrasonography
WBC: White blood cell count
DC: Disease controls
K2EDTA: Ethylenediaminetetraacetic acid dipotassium salt
APC-Cy7: AlloPhycoCyanine-cyanine7
PE-Cy7: PhycoErythrine- cyanine7
PE: PhycoErythrine
PerCP: Peridinin-chlorophyll-protein
IgG1: Immunoglobulin G1
PBS: Phosphate-buffered saline solution
DWI: Diffusion-weighted image
FLAIR: Fluid-attenuated inversion recovery
TR: Repetition time
TE: Echo time
TI: Inversion time
FOV: Field of view
NEX: Number of acquisitions
