# Supplementary figures and images for: Temporal changes in regulatory T cell subsets defined by the transcription factor Helios in stroke and their potential role in stroke-associated infection: a prospective case–control study
Source: J Neuroinflammation. 2023 Nov 23;20:275. doi: 10.1186/s12974-023-02957-w (PMC10666369; doi:10.1186/s12974-023-02957-w)

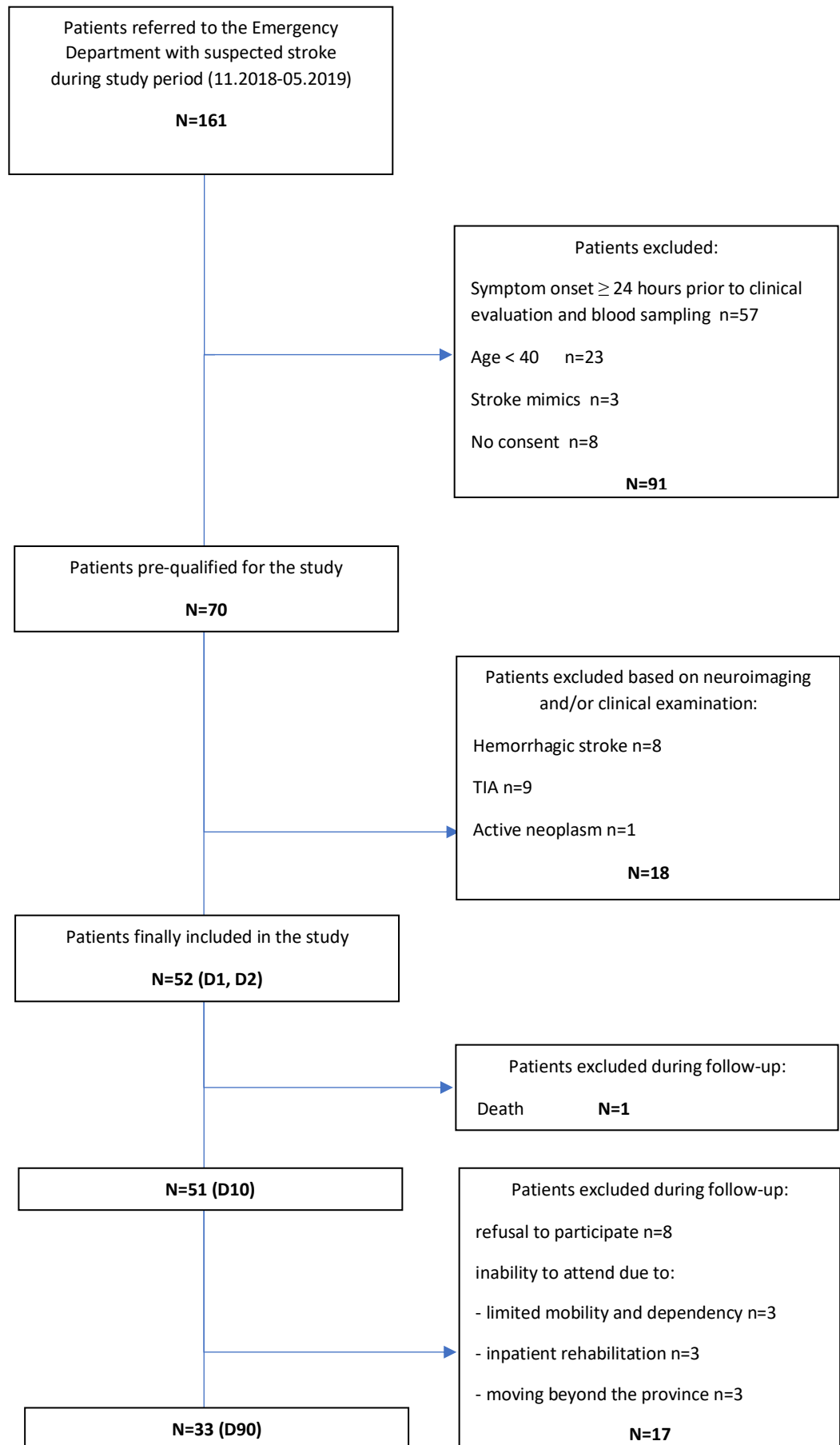

**Supplementary material S1.** Flow chart of participant recruitment

Supplement: Supplementary file 1 — Additional file 1: Flowchart of participant recruitment. [file 12974_2023_2957_MOESM1_ESM.pdf]
