# Supplementary material for: Temporal changes in regulatory T cell subsets defined by the transcription factor Helios in stroke and their potential role in stroke-associated infection: a prospective case–control study
Source: J Neuroinflammation. 2023 Nov 23;20:275. doi: 10.1186/s12974-023-02957-w (PMC10666369; doi:10.1186/s12974-023-02957-w)
Supplement: Supplementary file 2 — Additional file 2: Flow cytometry gating strategy. [file 12974_2023_2957_MOESM2_ESM.pdf]

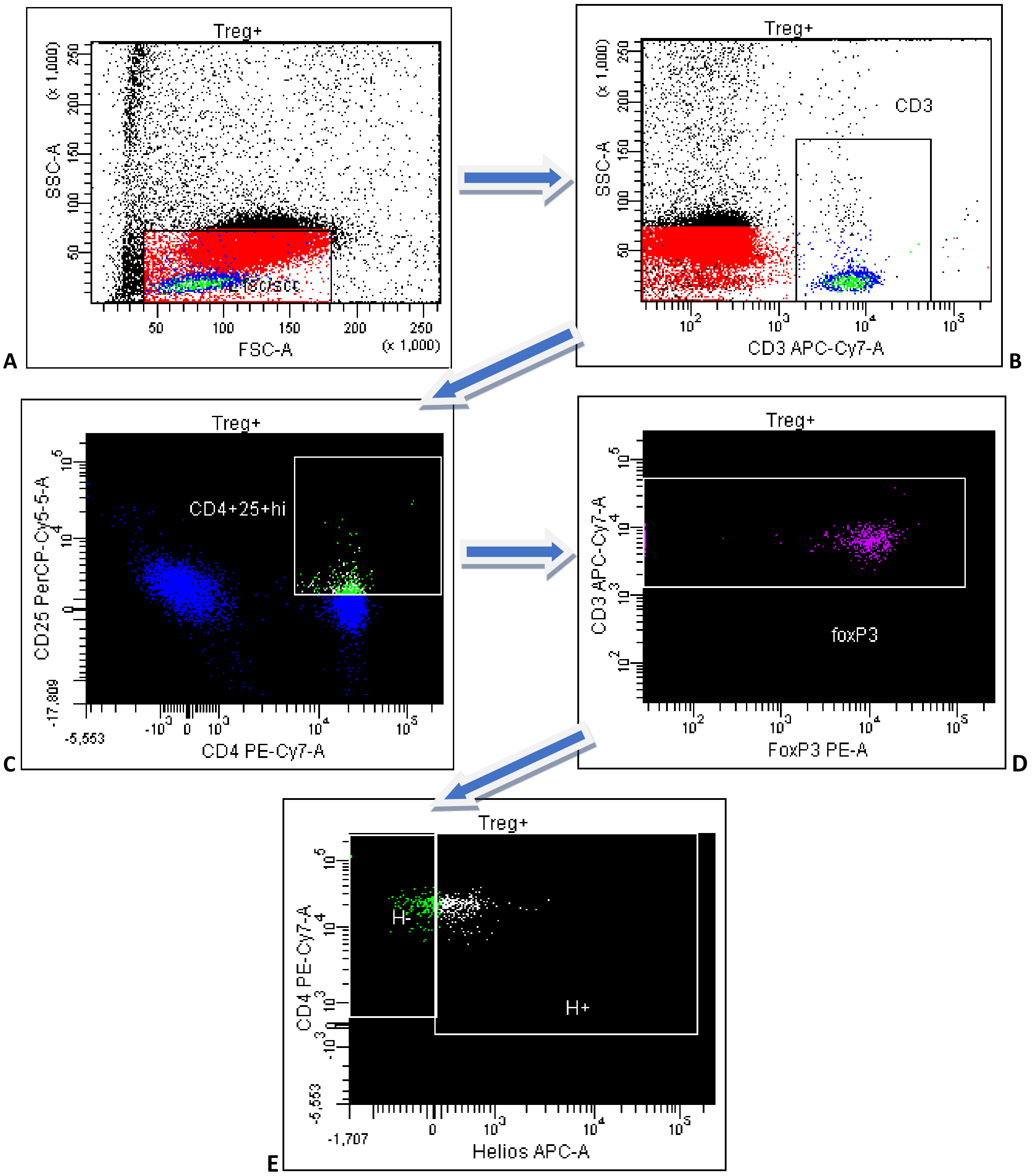

Supplementary Material S2 Flow cytometry gating strategy used to define Treg cells in the peripheral blood leukocytes. Lymphocytes were gated for expected morphology based on SSC-A versus FSC-A (A) and SSC-A/CD3^{+} (B). The regulatory T cell subpopulation was gated within the lymphocyte population based on the expression of CD4^{+}/CD25^{++} and FoxP3 (C+D). The Helios expression was assessed within the combined Tregs gates (E).
